# Nuptials captured in the time capsule

**DOI:** 10.1093/nsr/nwae261

**Published:** 2024-07-26

**Authors:** Shiqi Luo, Xin Zhou

**Affiliations:** Department of Entomology, China Agricultural University, China; Department of Entomology, China Agricultural University, China

Caddisflies (Insecta: Trichoptera) are one of the most speciose aquatic insect groups found in freshwater [1,2], and are widely applied in water quality monitoring [3]. The caddisflies share a behavioral trait, swarming—a large aggregation of conspecific individuals after emergence, with other common freshwater insects, such as mayflies, stoneflies, dragonflies, and basal dipterans. This behavior is considered as an adaptation to improve fitness, such as increasing mating chances. Swarming is observed in most extant caddisfly lineages but largely missing in a number of groups, such as the purse case makers (Hydroptilidae), tortoise case makers (Glossosomatidae), free-living caddisflies (e.g. Rhyacophilidae), and some tube case makers (e.g. Brachycentridae, Phryganeidae). Given the potential evolutionary advantage of swarming, when did it emerge in the caddisflies and why has it disappeared in some lineages? A recent study by Wang *et al.* [4] that integrates extant species and amber fossils has shed light on these intriguing questions.

The study [4] investigated six pieces of amber fossil from Myanmar, dated mid-Cretaceous. The researchers identified five new species from these ambers, each containing aggregated caddisfly adults of the same species. Morphological features well preserved in these ambers provided critical clues to support that the observed aggregation is a form of swarming (e.g. the sex ratio among the grouped adults and importantly, the mating positions captured between males and females).

To investigate the evolution of swarming behavior in caddisflies, this study created a phylogenetic tree using a multi-gene dataset from 16 families of extant caddisflies and placed the fossil species in this phylogeny using morphological traits. The ancestral state reconstruction revealed that swarming was already present in caddisflies when they first diverged from the lepidopterans (moths and butterflies), suggesting an ancestral trait. The presence and absence of swarming were mapped on the phylogeny, which indicated that these secondary losses were independent evolutionary events. Notably, the newly described purse case makers (Hydroptilidae) from the present study demonstrate swarming, which is absent in extant species of the family.

The study further investigated how predators have influenced the evolution of swarming behavior in caddisflies. By tracing the ancestral character of swarming in caddisflies and by bridging to the evolutionary timeline of potential predators and predatory behaviors, the study revealed that predation pressure might serve as the cause of secondary losses of swarming. The comparison among several potential predators suggested that bats may have played a significant role as the driving force.

In this study, Wang *et al.* [4] has established a set of criteria to identify swarming behavior in fossil insects. The integration of inferred behavior and phylogenetics has allowed the reconstruction of the evolutionary history of behavioral traits. Importantly, the fossil morphology provides novel and direct evidence, showing the presence of swarming behavior in certain caddisfly groups, that are otherwise missing in extant taxa. The addition of the fossil records has not only clarified the ancestral state of swarming for the focal lineage, but also pointed to the time period since which this behavior became lost, a timeline that coordinated nicely with the emergence of bats. This study demonstrates the great potential of integrating morphological and molecular evidence based on both extant and fossil samples, to infer the history of evolutionarily significant traits. By coupling fossil evidence with recent developments in insect phylogenomics [5–7], we anticipate that the understanding of trait evolution in the most diverse group of invertebrates will see unprecedented progress.
